# In Search of Wise Management of Medical Resources and Personnel in the Long Combat With Coronavirus

**DOI:** 10.3389/fsoc.2020.00040

**Published:** 2020-05-19

**Authors:** Yi-ru Hu, Mingqia Wang, Bin Zhang

**Affiliations:** PsyNI Lab, The Affiliated Brain Hospital of Guangzhou Medical University (Guangzhou Huiai Hospital), Guangzhou, China

**Keywords:** COVID-19, SARS-CoV-2, Medical resources, medical personnel, personal protective equipment (PPE)

It has been 5 months since the COVID-19 outbroke in Wuhan, China, at the end of 2019. Despite the small sigh of relief in China, the situation seems to be growing more intense in the rest of the world, as the number of cases of confirmed SARS-CoV-2 infection continues to increase. What is truly worrying is the condition of healthcare professionals. On March 25th, the number of medical personnel with confirmed infection had reached nearly 6,500 in Spain, representing almost 14% of the country's total cases, while in Italy, there was about 7,400, nearly one-tenth of its total cases (Wilson and Parra, 2020).

Besides the coronavirus, frontline healthcare workers are facing the threat of occupational burnout. Apart from taking care of a substantial number of patients with symptomatic COVID-19, healthcare providers are responsible for clinical screening for asymptomatic or minimally symptomatic patients with suspected COVID-19 while providing necessary health services for other non-infected patients. By April 7th, a doctor at the center of the largest outbreak in a county of the United States had cared for 450 people, among which 41 tested positive and three died. In a hospital in Chicago, the medical staff in an ICU need to do the job of doctors, nurses, and technicians (Fraser et al., 2020). Considering such a burden, any kind of health problems of healthcare workers, from mild mental stress to SARS-CoV-2 infection, may consequently have an impact on the already overloaded medical facilities, or worse yet, accelerate transmission of the pathogen, causing a more intractable situation.

Infection-control relies on the health system working at its best, and more essentially, depends on each individual frontline health professional working with adequate personal protective equipment (PPE), including masks, gowns, gloves, and eye protection. As far as we know, however, surging demand for PPE has become a major issue in different areas that has given rise to an interregional scramble for medical resources, potentially intensifying the stress of the disadvantaged areas (Durkee, 2020). The shortage of medical resources underlying the soaring demand for PPE means patients are receiving insufficient care and an increased risk of death, and increased exposure to the coronavirus for the non-infected. For the frontline workers, either way could bring additional workload and negative feelings including frustration, hopelessness, and self-accusing thoughts. Another problem resulting from such a shortage is inadequate self-protection of healthcare workers that led to concerns over their personal health and spreading the virus to families and friends. Thus, it is obvious that frontline workers are suffering from as much fear and anxiety as the public, as the shortfall of the healthcare workforce continues (Xiang et al., 2020).

## Joint Efforts in Guangdong

The COVID-19 pandemic is a global issue that no one can face alone. During the height of the epidemic, the Chinese government made every effort to curb outbreaks, mobilizing nationwide resources to support the fierce battle in Hubei, specifically in Wuhan (Chinese Center for Disease Control and Prevention, 2020; Guangming Daily, 2020). Substantial medical supplies such as PPEs and ventilators (a considerable proportion of which was donated by the public) were sent to Hubai, and more than 40,000 healthcare workers hailed from other parts of the country had been sent to help the situation. In addition, by early February, 2020, two Coronavirus hospitals and three mobile cabin hospitals had been available especially for patients with confirmed infections (Guangming Daily, 2020).

Meanwhile, Guangdong, the most crowded province in China with a population of 115.21 million (Statistics Bureau of Guangdong Province, 2020), was also struggling to survive the crisis that led to an escalating number of patients and an almost empty inventory of medical supplies. The situation became extremely urgent, with several major hospitals in Guangzhou (the capital city of Guangdong) pleading for public donation of PPEs on popular social media platforms in China, such as Weibo and WeChat (Chinanews, 2020).

The government acted quickly. Guangdong launched the first level response to major public health emergencies in late January (Health Commission of Guangdong Province, 2020b), while appealing for citizens to home quarantine and closely self-monitor their health conditions, going to hospital only when noticing COVID-19 symptoms or other circumstances that would require timely medical attention, in order to reduce the risk of hospital-acquired infections and help relieve stress on the healthcare facilities. Fortunately, the restrictions gained broad support, as people voluntarily stayed home despite the inconveniences caused.

Health workers have been getting the attention they deserve. A series of comprehensive, supportive policies for frontline medical staff, issued on February by the Guangdong government (Xinkuaibao, 2020), includes assistance on addressing problems regarding food, money, commuting, and other basic living needs, and suggesting separate work shifts for couples who are both frontline staff and responsible for children. Moreover, the government provided 24-h free mental health services and ensured accessible communication between frontline workers and their families, considering their mental stress during this hard time.

In response to the shortage of PPEs, the government called for a strengthening of the coordination of the production, supply, and distribution of medical supplies, ensuring the supply of important materials and giving priority to the needs of frontline medical staff and patients. They also established an online reporting system for inventory based on a comprehensive investigation of the stock and consumption; in addition, they established a system for the coordination of allocating supplies at the provincial and municipal levels to resolve the urgent shortages through centralized mobilization and emergency allocation (Southern Metropolis Daily, 2020).

The joint effort of the government and the public showed to be effective on flattening the curve in the province with over one hundred million people: by May 1st, the total number of confirmed infections in Guangdong was 1,588, with only eight cases of death (Health Commission of Guangdong Province, 2020a).

## Strategies on Supporting the Medical System

According to our experience in the past 4 months, it is vitally important to consider how to wisely collect and manage the medical resources during this challenging time, and it is crucial that available resources are made full use of wherever they are needed. It can be an option to address the shortages of medical supplies and workforces by facilitating international collaboration and interregional exchange or share of resources, experiences, and ideas, if possible.

Strategies regarding how to protect and preserve the healthcare workforces should be developed, with careful consideration of the roles for older personnel (Buerhaus et al., 2020). For example, flexible modification of the current shift work patterns in hospital settings may be a good way to ensure enough relaxation for the workers; if necessary, requesting retired health workers to consider returning to the workforce during the pandemic could be carried out, with their formerly direct clinical duties shifted to a supportive role with less exposure to the coronavirus, such as consulting, advising, and decision-making (Buerhaus et al., 2020).

In addition, offering accessible mental health services under the direction of expert psychotherapists or psychiatrists for workers in need should be considered. Such measures include but are not limited to: providing a safe, undisturbed space for rest and expressing concerns and emotions; helping to deal with negative feelings potentially associated with an unusual amount of deaths and critical patients; and providing information about how to identify and cope with the mental issues of patients or themselves, such as irritability, serious anxiety, or panic attacks.

All in all, it is crucial to ensure accessible resources work at their full capacity and to protect and support healthcare workers, in order to sustain the normal function of health systems all over the world in this crisis. Hopefully, more collaboration and coordination will help us bring the war to an early end.

## Author Contributions

YH, MW, and BZ have contributed to the conception, editing, and revision of the manuscript, approve the final version to be published, and agree to be accountable for all aspects of the work.

## Conflict of Interest

The authors declare that the research was conducted in the absence of any commercial or financial relationships that could be construed as a potential conflict of interest.
